# MiR-148a functions to suppress metastasis and serves as a prognostic indicator in triple-negative breast cancer

**DOI:** 10.18632/oncotarget.7953

**Published:** 2016-03-07

**Authors:** Xin Xu, Yun Zhang, Jeff Jasper, Erik Lykken, Peter B. Alexander, Geoffrey J. Markowitz, Donald P. McDonnell, Qi-Jing Li, Xiao-Fan Wang

**Affiliations:** ^1^ Department of Pharmacology and Cancer Biology, Duke University Medical Center, Durham, North Carolina, USA; ^2^ Department of Immunology, Duke University Medical Center, Durham, North Carolina, USA

**Keywords:** miR-148a, metastasis, triple-negative breast cancer, extravasation, prognosis biomarker

## Abstract

Triple-negative breast cancer (TNBC) presents a major challenge in the clinic due to its lack of reliable prognostic markers and targeted therapies. Accumulating evidence strongly supports the notion that microRNAs (miRNAs) are involved in tumorigenesis and could serve as biomarkers for diagnostic purposes. To identify miRNAs that functionally suppress metastasis of TNBC, we employed a concerted approach with selecting miRNAs that display differential expression profiles from bioinformatic analyses of breast cancer patient databases and validating top candidates with functional assays using breast cancer cell lines and mouse models. We have found that miR-148a exhibits properties as a tumor suppressor as its expression is inversely correlated with the ability of both human and mouse breast cancer cells to colonize the lung in mouse xenograft tumor models. Mechanistically, miR-148a appears to suppress the extravasation process of cancer cells, likely by targeting two genes WNT1 and NRP1 in a cell non-autonomous manner. Importantly, lower expression of miR-148a is detected in higher-grade tumor samples and correlated with increased likelihood to develop metastases and poor prognosis in subsets of breast cancer patients, particularly those with TNBC. Thus, miR-148a is functionally defined as a suppressor of breast cancer metastasis and may serve as a prognostic biomarker for this disease.

## INTRODUCTION

Breast cancer is the most commonly diagnosed and the second most fatal cancer in women. The current clinical treatment plan for breast cancer varies depending on characteristics of the primary tumor divided into four major molecular subtypes (LumA, LumB, Triple-negative/basal-like, and HER2 type) and two less common subtypes (Normal-like and Claudin-low) [1–3]. The triple-negative subtype of breast cancer, which often includes the Claudin-low subtype [4], lacks expression of estrogen receptor (ER), progesterone receptor (PR), and HER2, presents a particularly difficult therapeutic challenge as this subtype is characterized by its aggressive nature and lack of targeted therapies [5, 6]. The absence of tumor-specific treatment options for TNBC underscores the critical need to achieve a better understanding of the biology of this disease, as well as to identify better prognostic biomarkers and develop novel therapeutic strategies for those patients.

Metastasis is the main cause of cancer-associated death in many cancers, including breast cancer [7, 8]. Metastasis is a complex multistep process in which cancer cells migrate from the original tumor site to other parts of the body via the circulation system. In distal organs, tumor cells extravasate from the blood vessel and colonize the tissue to form metastatic lesions. Currently the metastatic potential of the primary breast tumor is estimated by pathological characterization of tumor grade and stage [9], and treatments for metastatic and earlier-stage breast cancer are very different. Therefore, identification of biomarkers to predict metastatic likelihood of the primary tumor can potentially be very useful to guide treatment for breast cancer.

MicroRNAs (miRNAs) are small non-protein-coding RNAs of ~22 nucleotides that negatively regulate gene expression via messenger RNA (mRNA) degradation or translational inhibition [10]. Due to its short seed region (2–7 nt), miRNAs often target multiple mRNAs simultaneously [11, 12]. Accumulating evidence strongly supports the notion that miRNAs play important roles in modulating all steps of the tumorigenic process [13, 14]. Particularly, multiple miRNAs have been shown to regulate metastasis through modulating the epithelial-to-mesenchymal transition, cancer cell invasion, the extracellular matrix or the tumor microenvironment [15–19]. In breast cancer, the expression profile or circulating levels of a number of miRNAs have been found to associate with the occurrence of metastasis and patient prognosis [20]. Several miRNAs, including miR-10b, miR-126, and the miR-200 family, have been identified as promoters or suppressors of breast cancer metastasis [21–25]. Since miRNAs tend to be more stable than mRNAs in formalin-fixed paraffin embedded tumor samples, probably due to their small sizes [26], they are good candidates to serve as biomarkers for diagnosis and prognosis estimation after pathologic examinations of biopsies [20, 27].

In the current study, we identified miR-148a as a suppressor of metastasis, particularly for TNBC, as its expression pattern is inversely correlated with tumor grade and metastatic potential, but directly correlated with prognosis of those patients since lower expression of miR-148 is associated with poor survival. Although miR-148a does not alter cell-autonomous behaviors including cell growth, viability, or migration *in vitro*, it suppresses cancer cell extravasation *in vivo*. Moreover, we identified WNT1 and NRP1 as potential targets of miR-148a involved in its tumor suppressive functions. Thus, miR-148a may be used as a prognostic biomarker for certain subtypes of breast cancer, including TNBC.

## RESULTS

### Low expression of miR-148a correlates with poor prognosis in breast cancer

**Figure 1:**
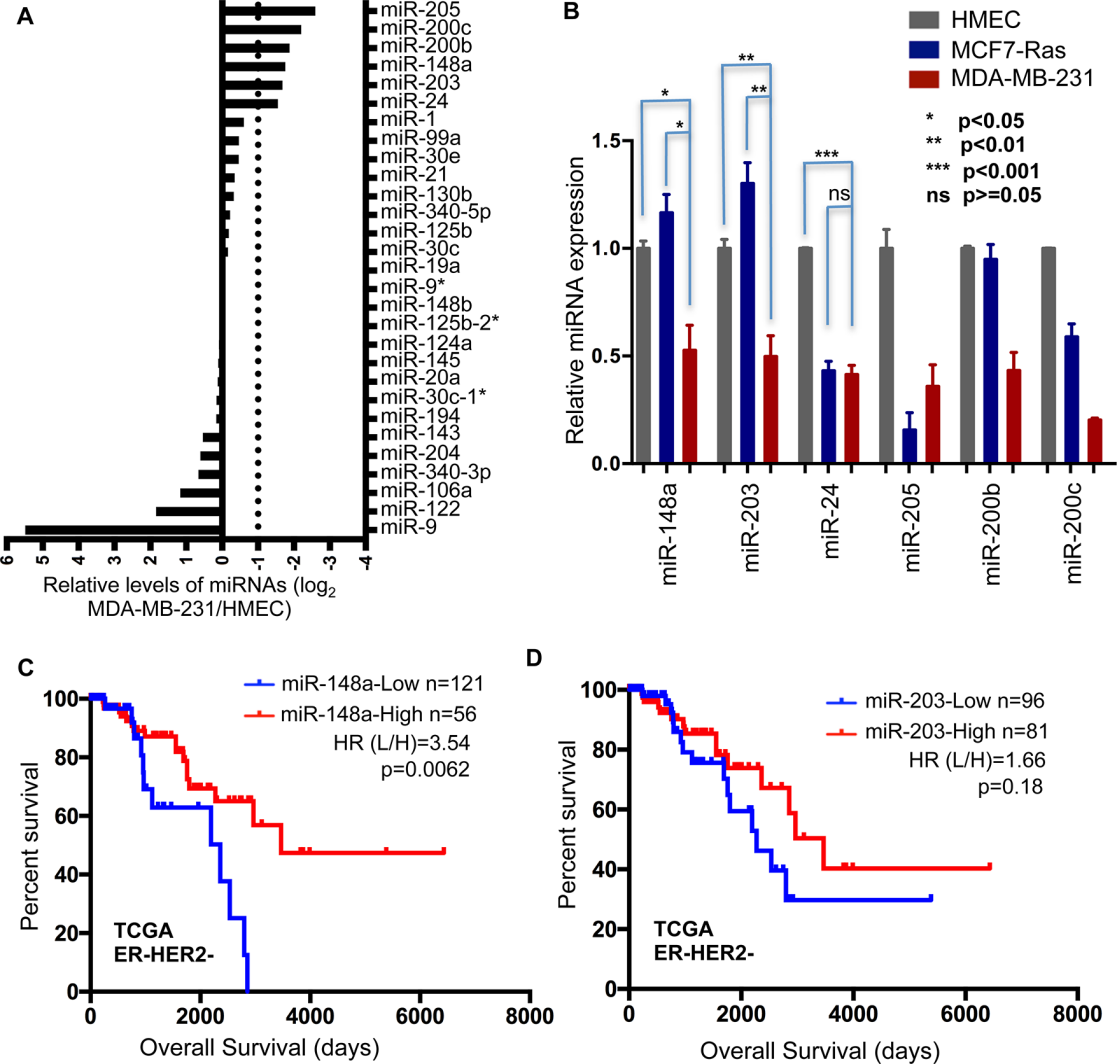
Low expression of miR-148a correlates with poor breast cancer patient prognosis (**A**) Expression levels of 29 miRNAs were profiled in MDA-MB-231 cells and normalized to levels in HMEC cells. (**B**) Expression of 6 miRNAs was determined in HMEC, MCF7-Ras, and MDA-MB-231 cells. Error bars indicate Standard Errors (*n* = 3). (**C**) and (**D**) Kaplan-Meier curves for overall survival for high and low expression of miRNAs, miR-148a (C) and miR-203 (D), in ER-HER2- subtype of breast cancer. Data was extracted from ***TCGA***. *P* values were calculated with Log-rank (Mantel-Cox) test. Hazard ratios were calculated using the method of Mantel-Haenszel.

To identify miRNAs involved in breast cancer metastasis, we analyzed miRNA gene expression profiles publically available in several databases for multiple cancers [28, 29]. Among the over 100 miRNA genes with altered expression in clinical samples, we selected a subset of 29 miRNA genes whose expression is down-regulated in metastatic tissues in comparison to non-metastatic tissues. A commonly used human breast cancer cell line with high metastatic potential, MDA-MB-231, was used to establish the baseline for expression levels of those candidate miRNAs. Since most of breast cancers arise from the mammary epithelium, we used HMEC (human mammary epithelial cells), a normal human breast cell line, along with MCF7-Ras cells, a tumorigenic but not metastatic cell line, as controls. Out of these 29 miRNAs detected at comparable levels, six miRNAs (miR-205, miR-200c, miR-200b, miR-148a, miR-203, miR-24) were found to be expressed at markedly lower levels in MDA-MB-231 cells compared to HMEC cells (top 6 miRNAs in Figure 1A). Four of these miRNAs, miR-148a, miR-203, miR-200b, and miR-200c, also displayed significantly reduced expression in the MDA-MB-231 cells compared to MCF7-Ras cells, suggesting that they may play roles in modulating metastasis (Figure 1B).

Since two of these miRNAs (miR-148a and miR-203) have not been previously reported to affect metastasis, we determined their clinical relevance by analyzing the survival time of patients with low or high levels of these miRNAs in the TCGA breast cancer patient database (Figure 1C, 1D, and Supplementary Figure 1). Considering that the metastatic cell line MDA-MB-231 is classified as representing TNBC, we paid extra attention to this subtype of breast cancer. Importantly, low expression of miR-148a was significantly associated with worse overall survival in patients classified as ER-negative HER2-negative (Figure 1C), which is consistent with our finding of reduced miR-148a level in the metastatic MDA-MB-231 cell line. In contrast, expression of miR-203 did not display a statistically significant association with patient prognosis in this cohort (Figure 1D), so we focused our study on the role of miR-148a in metastasis of breast cancer, in particular the triple-negative subtype.

### MiR-148a overexpression suppresses TNBC metastasis

**Figure 2:**
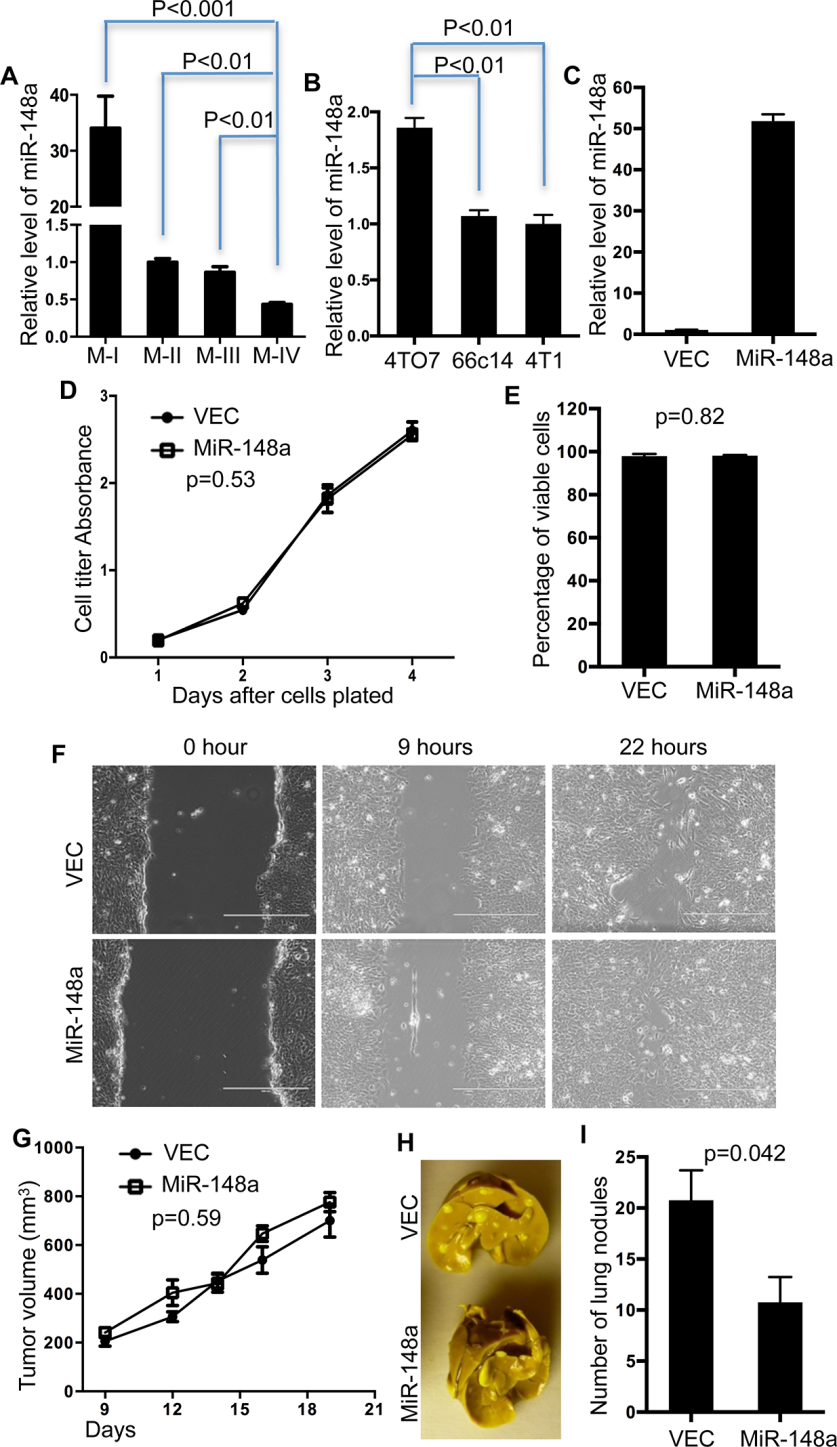
Overexpression of miR-148a suppresses 4T1 lung metastasis (**A**) Expression levels of miR-148a were determined in M-I, M-II, M-III, and M-IV cells and normalized to the expression level in M-II. Error Bars indicate Standard Errors (*n* = 3). (**B**) Expression levels of miR-148a were determined in 4TO7, 66c14, and 4T1 cells and normalized to the expression level in 4T1. Error Bars indicate Standard Errors (*n* = 3). (**C**) Expression levels of miR-148a were determined in 4T1 cells with overexpression of miR-148a (MiR-148a) normalized to control cells (VEC). Error Bars indicate Standard Errors (*n* = 3). (**D**–**F**) 4T1 cells with overexpression of miR-148a (miR-148a) and control cells (VEC) were examined for growth (D), viability (E), and migration/invasion (F). Error Bars indicate Standard Errors (*n* = 3). *P* values were calculated with two-way ANOVA (D) or unpaired, two-tailed *t* test (E). (**G**) Tumor sizes were measured and calculated at the indicated time points. *P* value was calculated with two-way ANOVA. Error Bars indicate Standard Errors (*n* = 5). (**H**) Representative pictures of lungs with metastatic nodules developed from 4T1-VEC or 4T1-miR-148a cells. (**I**) Lung nodules were quantified. Error Bars indicate Standard Errors (*n* = 5). *P* value was calculated with unpaired, two-tailed *t* test.

With expression of miR-148a in MDA-MB-231 cells being about 50% of its level in MCF7-Ras cells, we hypothesized that lower expression of miR-148a is correlated with TNBC metastasis. To test this, we utilized additional two series of mammary epithelial and TNBC cell lines and determined expression levels of miR-148a. The MCF10a series of cell lines, MCF10a-I, II, III, and IV, contains normal, tumorigenic but not metastatic, low metastatic potential, and high metastatic potential cell lines, respectively [30]. Consistent with our postulation, miR-148a expression gradually decreased in this series in accordance with increased metastasis potential (Figure 2A). We also examined miR-148a expression in the 4T1 series of murine breast cancer cell lines that also resemble TNBC cells. In this series, 4TO7 cells have the lowest metastatic potential, 66c14 cells have intermediate potential, while 4T1 cells have the highest metastatic potential [31]. Lower expression of miR-148a was detected in both 66c14 cells and 4T1 cells (Figure 2B). These data indicated that low expression of miR-148a is correlated with higher metastatic potential in multiple independent TNBC cell lines.

The 4T1 syngeneic mouse model has been widely used in studies of breast cancer metastasis as it closely mimics the pathological development of human breast cancer in a host with a competent immune system [32]. Since expression of miR-148a is down-regulated in 4T1 cells, we ectopically expressed miR-148a in 4T1 cells by viral infection (Figure 2C) and determined the effects of this manipulation on various aspects of cancer development. The cell-autonomous properties, including cell growth, viability in culture, and *in vitro* migration ability, of 4T1 cells with ectopically elevated miR-148a were indistinguishable from control 4T1 cells in these three aspects (Figure 2D, 2E, and 2F). We next implanted 4T1 cells with or without miR-148a overexpression into the mammary fat pad of female Balb/c mice, and found that primary tumors formed and progressed with similar kinetics (Figure 2G). Finally, we analyzed the impact of miR-148a on lung metastasis by counting macroscopic tumor nodules on the surface of the lungs and found approximately half as many tumor nodules with 4T1 cells overexpressing miR-148a compared with control 4T1 cells (Figure 2H and 2I). Therefore, miR-148a suppresses lung metastasis of 4T1 cells in this syngeneic mouse model.

### Overexpression of miR-148a alters cancer cell propagation *in vivo*

**Figure 3:**
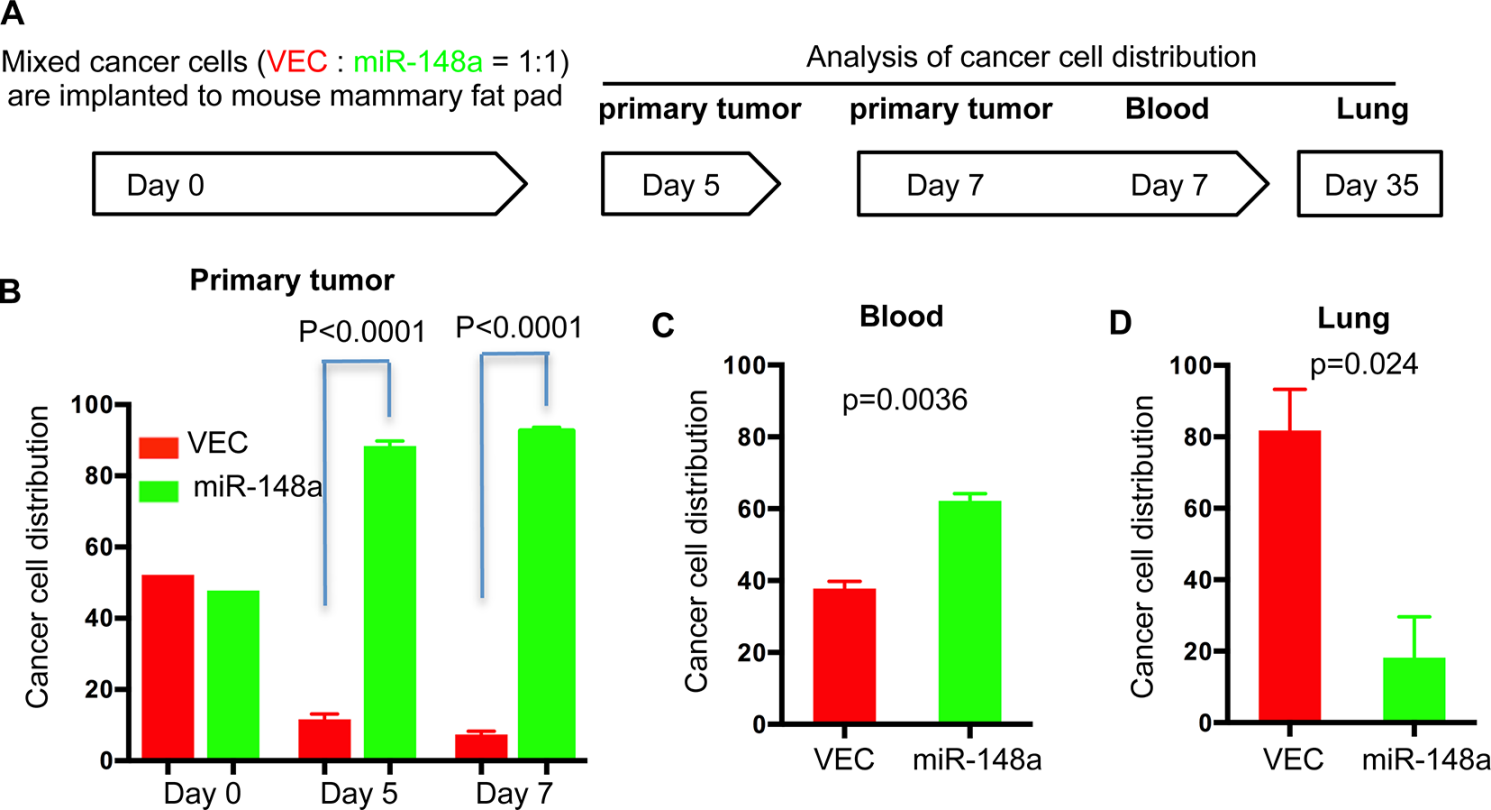
Overexpression of miR-148a alters 4T1 cancer cell propagation *in vivo* (**A**) Illustration of the procedure for tracking cancer cell propagation *in vivo*. 4T1 control cells with expression of iRFP (VEC, red) and 4T1cells with expression of miR-148a and GFP (miR-148a, green) were mixed and implanted into the mammary fat pad of Balb/c mice. Mice were sacrificed and tissues were harvested at the indicated times. Tissues were digested followed by analysis using flow cytometry. Cancer cells with expression of iRFP or GFP were quantified as the percentage of the total number of cancer cells in each sample. (**B**) Distribution of cancer cells in primary tumors at day 5 and day 7. Quantification of cancer cells at day 0 is from mixed cells prior to implantation. (**C**) Distribution of cancer cells in blood at day 7. (**D**) Distribution of cancer cells in lungs at day 35. Error bars are Standard Errors (*n* = 5). *P* values were calculated with paired, two-tailed *t* test.

It has been reported that the tumor microenvironment contains many types of non-cancer cells that regulate cancer development and immune response [33, 34]. In our study, we also noticed that cancer cells account for only 5–10% of the cells in the primary tumor formed by 4T1 cells at early time points post-implantation, and the percentage of cancer cells continues to decrease as the tumor progress. Therefore, although the macroscopic parameters of the primary tumors that arose from 4T1 cells with overexpression of miR-148a are similar to those of tumors derived from control cells, it is possible that miR-148a affects cancer cell propagation *in vivo* without presenting noticeable differences in tumor size. To test this possibility, we labeled control 4T1 cells with iRFP (marked as red for convenience) and miR-148a-overexpressing cells with GFP (green) for engraftment in the same mouse with easy detection and quantification of different cells via flow cytometry. This approach enabled us to track these cells and rule out intra-mouse variations, such as primary tumor immune infiltration, which could have a significant effect given the large portion of the primary tumor mass made up of non-transformed cells. We first confirmed that expression of miR-148a was similar to that achieved in previous experiments (Supplementary Figure 2A), and that relative cell growth of the lines *in vitro* when mixed 1:1 was similar at multiple time points, indicating that miR-148a overexpression did not alter relative cell growth compared to control (Supplementary Figure 2B). 4T1-miR-148a and 4T1-VEC cells were then mixed at a 1:1 ratio and implanted into the mammary fat pad of Balb/c mice. Analysis was performed following the schematic illustrated in Figure 3A. Up until 5 days after implantation, the inoculated 4T1 cells did not form a solid primary tumor mass in the mammary fad pad, so we analyzed cancer cell presence in the primary tumors at day 5 and day 7 after tumor cell implantation. We also examined the blood for the presence of circulating tumor cells at day 7 since this is the time point at which we could observe circulating tumor cells in this model.

At day 5, 4T1-miR148a cells dominate the primary tumor, constituting about 80% of the total cancer cell population. At day 7, the presence of 4T1-miR-148a remained dominant, with a slightly increased presence (Figure 3B). These tumor cells were indeed alive, as assessed by flow cytometry following PI exclusion staining of dissociated tumor cells. To rule out the possibility that the observation was due to different immune response to fluorescent proteins, we used GFP for vector control and BFP for miR-148a overexpression. Consistently, the presence of 4T1-miR-148a remained dominant (Supplementary Figure 2C). There were also more 4T1-miR-148a cells than control 4T1 cells in the blood at day 7, although the disparity was not as great as in the primary tumors (Figure 3C). Although 4T1-miR-148a cells were more abundant in the blood, they occupied a smaller proportion of the total cancer cells in the lung, comprising less than 20% of cancer cells in that tissue (Figure 3D). These results suggest that the extravasation process, in which circulating cancer cells penetrate the endothelial barrier of blood vessels and gain access to the lung, may be suppressed by miR-148a.

### Manipulation of miR-148a expression alters lung colonization of breast cancer cells

**Figure 4:**
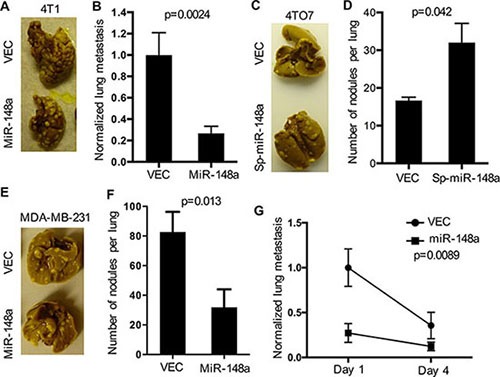
Overexpression of miR-148a regulates cancer cell lung colonization (**A**–**F**) The lung colonization ability of cancer cells with manipulated levels of miR-148a and control cells were determined by quantification of lung nodules raised from cancer cells administered directly into the blood. Error bars are standard errors (*n* = 5). *P* values were calculated with unpaired, two-tailed *t* test. (A) and (B) 4T1 cells with over expression of miR-148a and control cells. Representative picture (A) and quantification (B) are shown. (C) and (D) 4TO7 cells with down-regulation of miR-148a and control cells. Representative picture (C) and quantification (D) were shown. E and F, MDA-MB-231 cells with over expression of miR-148a and control cells. Representative picture (E) and quantification (F) are shown. (**G**) Analysis of cancer cell extravasation at day 1 and day 4 after administration. Cancer cells that underwent extravasation into lung were quantified by flow cytometry and normalized to 4T1 control cells. Error bars are standard errors (*n* = 3). *P* values were calculated with two-way ANOVA.

To directly test whether miR-148a suppresses extravasation of 4T1 cells *in vivo*, we administrated cancer cells directly into the blood system of the mouse through tail vein injection, and subsequently examined lung colonization. 4T1 cells with overexpression of miR-148a formed fewer colonies in the lungs than control 4T1 cells (Figure 4A). Quantification of tumor burden by qRT-PCR also demonstrated that miR-148a overexpression caused a dramatic decrease in the lung colonization of cancer cells (Figure 4B). To further examine whether decrease in miR-148a level is a major factor promoting cancer cell metastasis, we manipulated expression of miR-148a in cancer cells with low metastatic potential and examined the lung metastasis of these cells. 4TO7 cancer cell line is well-known to have a low metastatic potential, as well as having a higher expression of miR-148a than the more metastatic 4T1 cell line, and is thus ideal for probing this issue (Figure 2B and [31]). We utilized a microRNA sponge targeting miR-148a to decrease its expression in 4TO7 cells (Supplementary Figure 3). Consistent with the suppressive function of miR-148a in cancer cell lung colonization, decreasing in miR-148a promoted lung colonization of 4TO7 cells when they were inoculated intravenously (Figure 4C and 4D).

We also tested if our findings on the suppressive effects of miR-148a on metastasis in the mouse cell lines were applicable to human cells. We first overexpressed miR-148a in MDA-MB-231 cells (Supplementary Figure 4A), and evaluated their cell-autonomous behaviors, including cell growth, viability in culture, and migration. Similar to the results found in 4T1 cells, overexpression of miR-148a did not alter cell-autonomous characteristics of MDA-MB-231 cells (Supplementary Figure 4B–4F). Moreover, overexpression of miR-148a did not affect primary tumor formation by MDA-MB-231 cells (Supplementary Figure 4G), but reduced lung colonization after their administration into the venous system of nude mice (Figure 4E and 4F). Cancer cell lung colonization requires extravasation, seeding, and expansion. Based on the observation that overexpression of miR-148a did not alter tumor nodule size in the lung (Figure 4A, 4C, and 4E), we tested more directly if extravasation was altered. It has been reported that approximately 75% of cancer cells which survive in the blood circulation could extravasate by 24 hours after their intravasating [35]. Therefore, we examined cancer cell presence in the lung at day 1 and day 4 after inoculation of cancer cells. Interestingly, overexpression of miR-148a caused at least a 50% reduction in the numbers of MDA-MB-231 cells present in the lung (Figure 4G), which supported our hypothesis that miR-148a likely suppresses cancer cells extravasation.

### Identification of WNT1 and NRP1 as potential target genes of miR-148a

**Figure 5:**
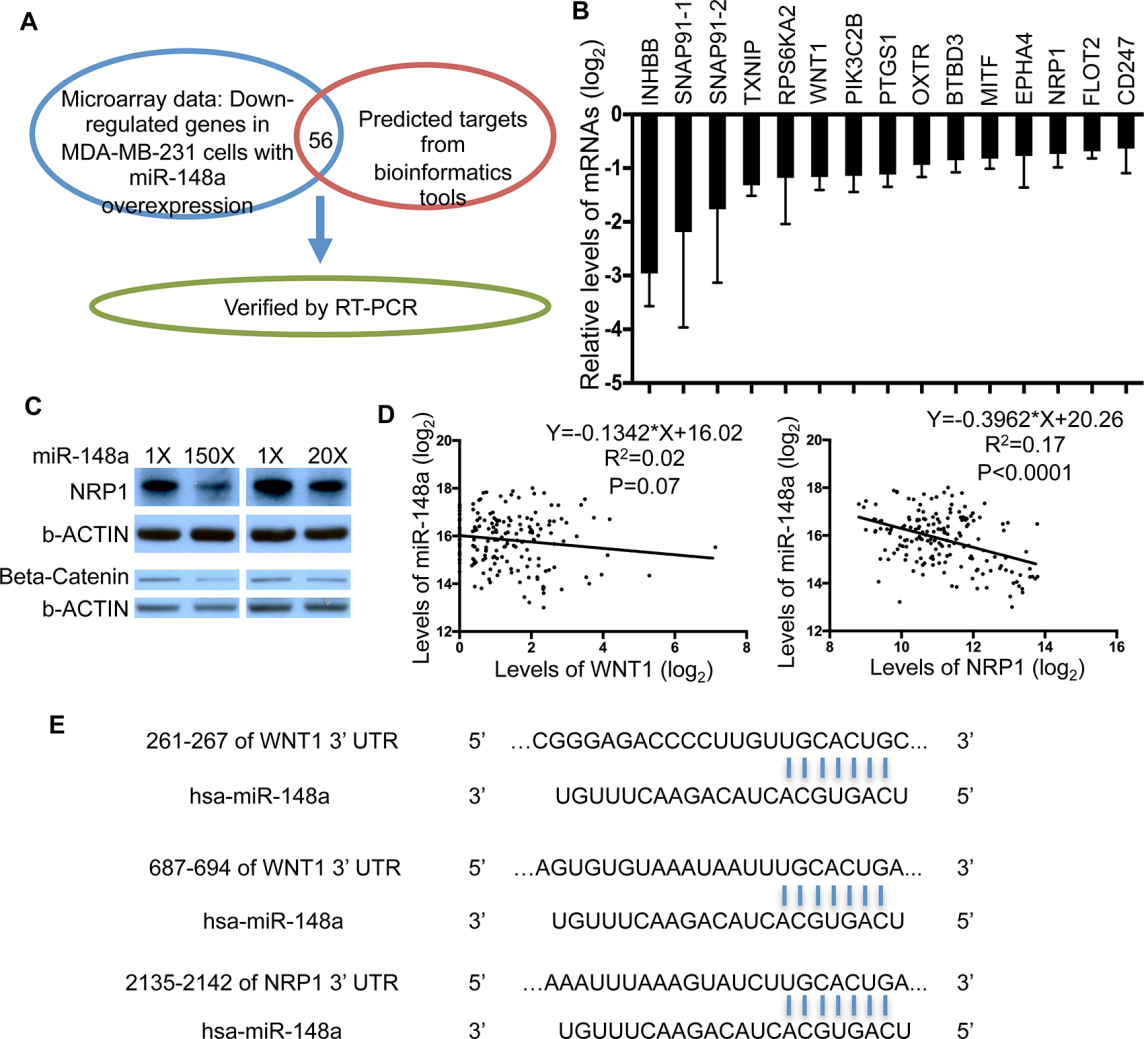
Identification of WNT1 and NRP1 as target genes of miR-148a in MDA-MB-231 cells (**A**) Illustration of strategy used to identify target genes of miR-148a. (**B**) qRT-PCR analysis was performed to validate mRNA levels altered by overexpression of miR-148a. (**C**) Protein abundance of WNT1 and NRP1 altered by miR-148a were determined by Western Blot. Two levels of miR-148a overexpression (150× and 20×) were achieved in MDA-MB-231 cells. (**D**) Correlation analysis with linear regression between levels of miR-148a and its target genes was done with GraphPad Prism. The expression levels were extracted from the TCGA database. (**E**) The 3′-UTR of the WNT1 and NRP1 genes contain binding sites for miR-148a seed region according to the TargetScan prediction program.

To investigate how miR-148a suppresses breast cancer metastasis, we took a systematic approach to identify target genes of miR-148a in MDA-MB-231 cells (Figure 5A). Although alteration of mRNA level is not the only mechanism by which miRNAs regulate gene expression, detectable mRNA alteration can be observed with enhanced overexpression of miRNAs most of time [36]. Therefore, we performed a microarray analysis to determine mRNA level alterations in an unbiased fashion in MDA-MB-231 cells with enhanced overexpression of miR-148a. Only genes with mRNA levels decreased by at least 20% were selected for further validation as possible target genes of miR-148a. Hits from the microarray were subsequently overlapped with two algorithms used to predict potential miRNA targets, Targetscan and Miranda, to narrow down candidates, resulting in 56 genes as potential targets. Quantitative RT-PCR was performed to validate these mRNA alterations by enhanced overexpression of miR-148a. Genes with a “ΔΔCt < −0.5 are shown in Figure 5B.

Our previous results demonstrated that miR-148 does not strongly influence MDA-MB-231 cell-autonomous behaviors. One possible explanation for this observation is that miR-148a regulates genes encoding proteins secreted or localized to the outer plasma membrane and interacting with the tumor microenvironment to suppress cancer metastasis. Therefore, we searched for known functions and subcellular localizations of our candidate genes, and subsequently focused on those with the expected properties (Supplementary Table 1). Theoretically, levels of target genes in human breast cancers should inversely correlate with miR-148a expression. Through correlation analysis of gene expression profiles between candidate genes and miR-148a in the TCGA breast cancer patient database, we further narrowed down targets of miR-148a to four genes, INHBB, PIK3C2B, WNT1 and NRP1. Previous studies have shown that INHBB acts to suppress tumorigenesis and targeting PIK3C2B impairs cell proliferation in a cell-autonomous manner [37, 38], functions that are inconsistent with the theoretical characteristics of miR-148a target genes to modulate tumor microenvironment in our experimental setting. On the other hand, several previous studies have implicated NRP1 and WNT signaling in various types of cancer growth and progression [39–41], which are consistent with the expected characteristics of miR-148a target genes. Therefore, we focused on validating NRP1 and WNT1 as targets of miR-148a in this study. Expression of the WNT downstream signaling effector β-catenin was used to measure the activity of WNT1 in this study due to the technical difficulty of detecting WNT1 protein by Western blotting. The reduction of β-catenin and NRP1 proteins was confirmed in MDA-MB-231 cells with two different levels of miR-148a overexpression (Figure 5C). In addition, correlation analysis between expression profiles of these mRNAs and miR-148a in TCGA database showed that expression of WNT1 and NRP1 is inversely correlated with levels of miR-148a (Figure 5D). Furthermore, the 3′-UTRs of both NRP1 and WNT1 contain perfect binding sites for miR-148a (Figure 5E), as predicted by the TargetScan algorithm. Taken together, these results indicate NRP1 and WNT1 as molecular targets of miR-148a with biological functions relevant to TNBC metastasis.

### MiR-148a is a potential biomarker for prognosis of breast cancer patients

**Figure 6:**
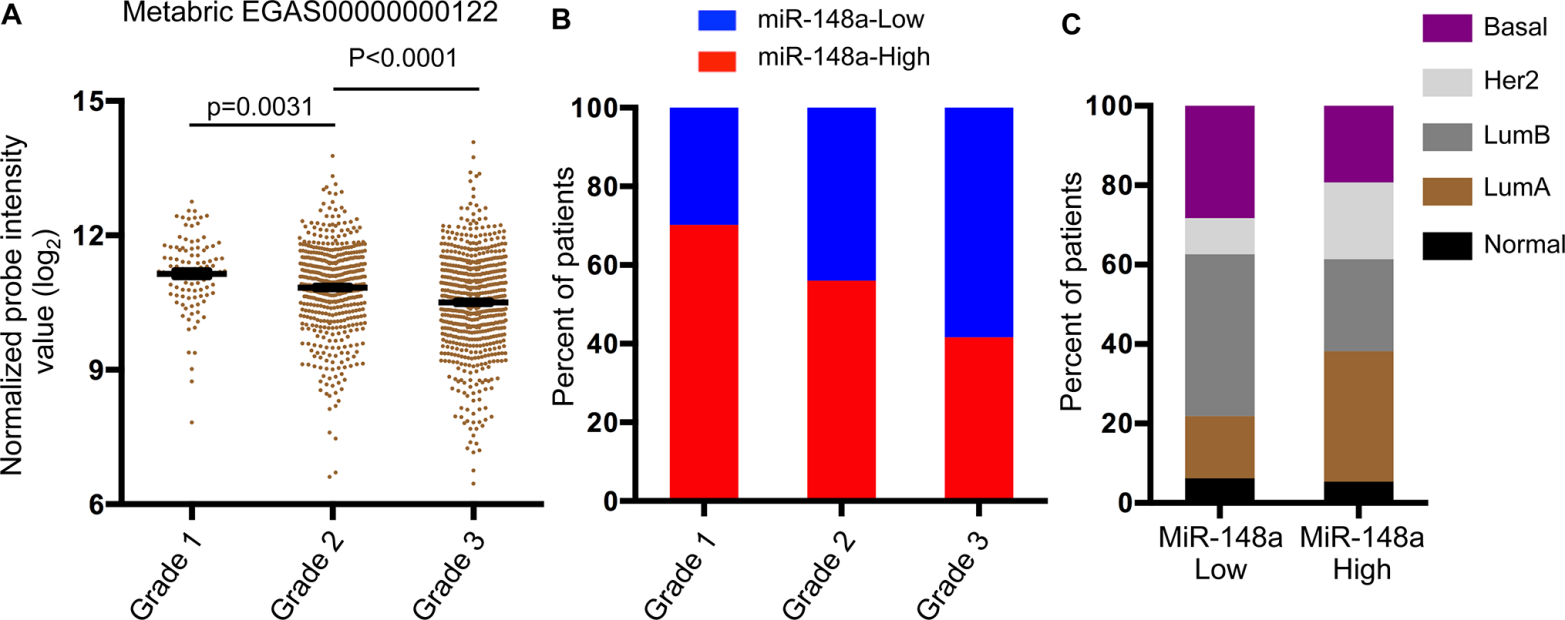
Low expression of miR-148a is associated with worse diagnosis of breast cancer patients Data was extracted from ***METABRIC data*** [55] (**A**) miR-148a expression was analyzed in patients stratified by tumor grade. Error bars are standard errors (grade 1, *n* = 104; grade 2, *n* = 496; grade 3, *n* = 623). *P* values were calculated with unpaired, two-tailed *t* test. (**B**) Patients were categorized as high or low expression of miR-148a and percentage of patients with high or low expression of miR-148a was calculated in patients stratified by tumor grade. (**C**) Percentage of patients within each breast cancer subtype was calculated in patients categorized by high or low expression of miR-148a in primary tumors.

Previously we demonstrated that TNBC patients with low expression of miR-148a tend to have a worse prognosis (Figure 1C). To determine whether miR-148a can be used as a potential biomarker for prognosis estimation, we analyzed the Metabric breast cancer patient database, which contains patient status with tumor grade and metastasis. During clinical diagnosis, histologic tumor grade is an important factor considered during determining the appropriate treatment plan for breast cancer patients [9], as patients with higher-grade tumors tend to have a worse prognosis. Through statistical analysis, we found that the higher the grade of the primary tumor, the lower the expression of miR-148a (Figure 6A). In addition, we quantified the percentage of patients with low or high expression of miR-148a in each grade. Patients with low miR-148a in the primary tumor occupied 31% of the pool in grade 1, 44% in grade 2, and about 60% in grade 3 (Figure 6B). Finally, we analyzed the expression of miR-148a in different subtypes, and found that patients with either Basal or Luminal B subtype primary tumors are more likely to be classified as having low miR-148a expression (Supplementary Table 2 and Figure 6C).

**Figure 7:**
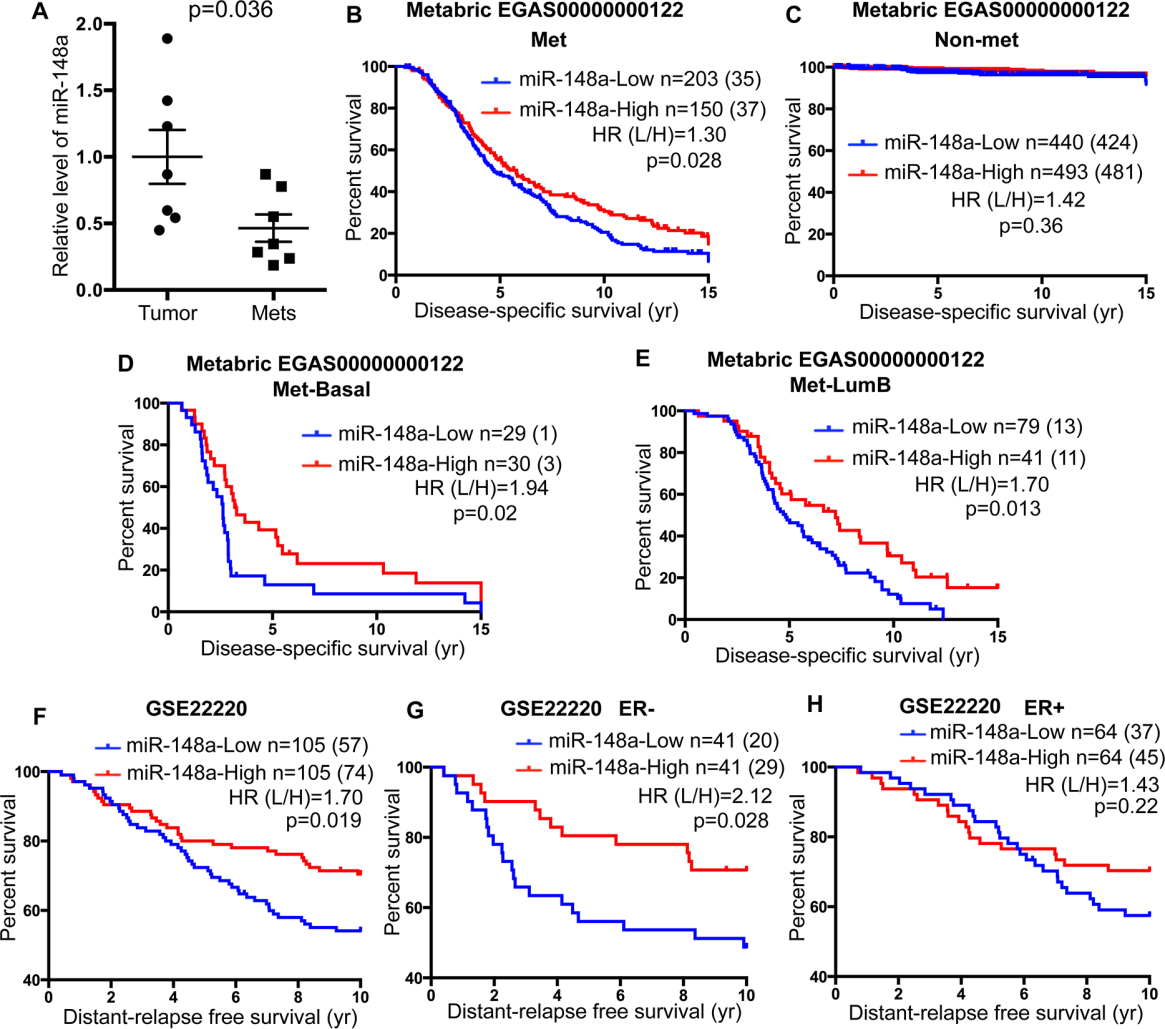
Low expression of miR-148a is associated with poor prognosis of breast cancer patients (**A**) Levels of miR-148a expression were determined in primary (Tumor) and metastatic (Mets) tumor samples from breast cancer patients [19] and normalized to the levels of miR-148a in primary tumors. Error bars are standard errors (*n* = 7). *P* value was calculated with unpaired, two-tailed *t* test. (**B**–**E**) Kaplan-Meier curves for disease-specific survival for high and low expression of miR-148a in breast cancer patients with metastasis (B) or without detectable metastasis (C). Basal subtype (D) and LumB subtype (E) of patients with metastasis were further analyzed using Kaplan-Meier curves for high and low expression of miR-148a. Data was extracted from ***METABRIC data*** [55]. Survival time was cropped at 15 years. (**F**–**H**) Kaplan-Meier curves for distant-relapse free survival for high and low expression of miR-148a in total breast cancer patients (F), ER-negative patients (G), and ER-positive patients (H). Data was extracted from **GSE22220**. B-H, *P* values were calculated with Log-rank (Mantel-Cox) test. Hazard ratios were calculated with the method of Mantel-Haenszel).

Higher histologic tumor grade is associated with high tumor metastatic potential [9]. We therefore further analyzed multiple breast cancer patient databases annotated with both miRNA expression and metastatic status, and discovered that primary tumors from patients who were diagnosed with detectable distant metastasis have lower expression of miR-148a than primary tumors from patients without metastasis (Supplementary Table 3). Furthermore, we determined miR-148a expression level in patient tissue samples from primary tumors and metastasis. The expression of miR-148a was significantly decreased in metastatic tissue (Figure 7A). To determine whether expression of miR-148a in the primary tumor and presence of distal metastasis are directly associated with patient outcome, we first correlated disease-specific survival with expression of miR-148a in patients with or without metastasis separately. Consistently, lower expression of miR-148a in the primary tumor was correlated with shorter survival for patients with metastasis but not for patients without metastasis (Figure 7B and 7C). By analyzing different subtypes of patients with metastasis, we found that the expression of miR-148a was significantly correlated with survival time only for patients with Basal or Luminal B subtype primary tumors (Figure 7D, 7E, and Supplementary Figure 5A–5C).

To directly determine whether the expression level of miR-148a is associated with metastatic development in breast cancer patients, we also analyzed a patient database annotated with time from initial diagnosis to metastasis development (GSE22220). Interestingly, the expression of miR-148a is significantly associated with the time to develop metastasis (Figure 7F). Moreover, the correlation of miR-148a expression to metastatic development occurs in ER-negative patients but not ER-positive patients (Figure 7G and 7H). Taken together with the correlation of miR-148a expression to survival time of patients with metastasis, these results indicate that miR-148a might serve as a potential prognostic marker for TNBC patients.

## DISCUSSION

In this study, we show that miR-148a is down-regulated in breast cancer cell lines with high metastatic potential. Although overexpression of miR-148a did not alter cell-autonomous behaviors *in vitro*, it suppressed breast cancer metastasis *in vivo*, achieved mainly through reducing cancer cell extravasation. Using statistical analysis of multiple databases of breast cancer patients, we found that low expression of miR-148a is associated with diagnosis of high-grade primary tumors and poor prognosis of breast cancer patients, particularly for patients with Basal and Luminal B subtypes. Therefore, we propose that the expression status of miR-148a could serve as a molecular biomarker for the diagnosis of primary tumors in breast cancer patients.

Deregulation of miR-148a has been reported in several types of cancer, including non-small-cell lung cancer, osteosarcoma, gastric cancer, ovarian cancer, glioblastoma, pancreatic cancer, bladder cancer, hepatocellular carcinoma, and breast cancer [42–46]. In addition, miR-148a has been previously characterized as a tumor suppressor with functions in regulating cell proliferation, apoptosis, and migration/invasion, all of which are cell-autonomous behaviors [43, 47–49]. In contrast to these reports, we did not detect cell-autonomous alterations mediated by miR-148a in TNBC *in vitro*. Instead, our results indicate that miR-148a likely suppresses cancer cell extravasation *in vivo*, which requires interactions with the tumor microenvironment. Furthermore, after co-implantation of 4T1 cells into the mammary fat pad of recipient mice, we discovered that cancer cells with overexpression of miR-148a dominate the proportion of cancer cells in the primary tumor compared with control cancer cells (Figure 3B). Consistently, no correlation between patient tumor size and expression of miR-148a was observed (Supplementary Figure 6). It will be interesting to test in the future whether the proliferation and viability of 4T1 cells are altered by miR-148a *in vivo* through changing the interactions between cancer cells and microenvironment.

As discussed earlier, although many targets of miR-148a have been identified in various cancers, they mainly function in regulating cancer cell-autonomous behaviors [43, 47–49]. In addition, miR-148a has been reported to suppress angiogenesis through down-regulation of ERBB3 and IGF1R [50, 51]. In our experimental setting, miR-148a did not appear to affect angiogenesis at the primary tumor site (Supplementary Figure 7), although we cannot rule out the possibility that miR-148a could suppress neo-angiogenesis in the lung during colonization. In agreement with this observation, mRNA abundance of ERBB3 or IGF1R was not affected by miR-148a in our microarray analysis. Instead, we made the finding that in our system miR-148a targets WNT1 and NRP1. WNT signaling has been reported to regulate the epithelial-mesenchymal transition, which is well known to promote cancer cell metastasis in a variety of settings [7, 39]. Consistent with this, our results show that miR-148a suppresses breast cancer metastasis possibly through down-regulation of WNT1. NRP1 has recently been identified as a target gene of miR-148a in medulloblastoma [52]. In breast cancer, several studies have implicated NRP1 in various aspects of cell growth, survival, migration, and metastasis [53, 54]. Consistent with our results, NRP1 functions to protect cancer cells from stress-induced apoptosis and mediates their spreading and membrane ruffling *in vitro* [55], which are crucial processes in metastasis.

Our discovery indicates that miR-148a suppresses breast cancer metastasis in multiple xenograft mouse models. It has been reported in several studies that miRNAs can be used as therapeutic agents for cancer in mouse models [56]. Together with statistical analyses demonstrating that miR-148a may serve as a prognostic biomarker for TNBC metastasis, further efforts should be made to investigate whether administration of miR-148a could be therapeutically efficacious to suppress the metastasis of breast cancer.

## MATERIALS AND METHODS

### Cell culture

MDA-MB-231 (ATCC Cat. HTB-26), 4T1, 4TO7, 66c14 cells were cultured in DMEM supplemented with 10% FBS and 1% penicillin/streptomycin. The 4TO7, 66c14, and 4T1 cell lines were kindly provided by Dr. Yibin Kang at Princeton University. HMEC cells (ATCC Cat. PCS-600-010) were cultured in MEGM (Lonza CC-3151) supplemented with MEGM SingleQuots (Lonza CC-4135). MCF7-Ras cells were cultured in DMEM/F-12 supplemented with10% FBS and 1% penicillin/streptomycin. MCF10 and derived cell lines were kindly provided by Dr. Lalage M. Wakefield at the National Cancer Institute and cultured as described in [30]. All cells were cultured at 37°C, 5% CO_2_.

### RNA extraction and real-time PCR

The total RNA of cells was isolated with Trizol (Life Technology) according to the manufacture's instruction. We used SYBR-based RT-PCR to quantify mature miRNA and mRNA expression. To quantify miRNA, *Escherichia coli* polyA polymerase was employed to add adenines at the 3′ end of RNA molecules lacking a poly A tail. Oligo(dT) immediately followed by a universial tag was used to synthesize the cDNA by superscript III reverse transcriptase (Invitrogen). Then qPCR was performed with this universal tag and miRNA-specific forward primers in a pre-mixed PCR reaction (Quanta Biosciences). To quantify mRNA, total RNA was used to synthesis cDNA with iScript (Bio-Rad) followed by qPCR with forward and reverse primers.

### Clinical samples

The tumor specimens and tissue samples from patients used in this study were described as [22].

### Plasmids

Plasmid DNA used for ectopic overexpression of miR-148a was constructed by inserting synthesized pri-miR-148a into pMSCV-Puro vector (Clontech) either alone or together with GFP open reading frame. MiR-148a sponge was designed as [57]. It contains 10 repeats of DNA fragment-5′ ACAAAGTTCT VBHTGCACTGA- into pMSCV-Puro vector immediately after GFP open reading frame.

### Cell growth/viability assay

Cell growth was determined with CellTiter-Glo Luminescent Cell Viability Assay (Promega). Cell viability was determined by PI exclusion followed by flow cytometry. For cell viability in suspension culture, cells were cultured on Ultra Low Attachment plates (Corning) followed by PI exclusion cell viability assay.

### Migration/scratch assay

Migration assay was performed with 6-well trans-well chamber (Corning). Cells were added in the upper chamber with serum free medium and medium with 10%FBS was loaded in the bottom chamber as attractant. Membranes were processed following standard protocols. Migrating cells were fixed with 4% formaldehyde and stained with 1% Crystal Violet. For scratch assay, cells were plated to achieve 100% confluence on plates. Scratches were generated by tips and the wound healing was monitored under microscope following standard protocols.

### Microarray

Microarray profiling was performed with GeneChip Human Genome U133A 2.0 (Affymetrix, Inc., 900468) and raw data was processed and analyzed by Duke University Microarray Core Facility.

### Western blot

For protein quantification, total cell extracts were fractioned by electrophoresis on SDS-PAGE followed by transferring onto a PVDF membrane. Western blotting was performed with the following primary antibodies: anti-beta Catenin (ZYMED, 13-8400), anti-NRP1 (Santa Cruz Biotechnology, sc-7239), and anti-beta-actin (Cell Signaling, 3700S).

### Animal studies and tissue analysis

All research involving animals complied with protocols were approved by the Duke University Animal Care and Use Committee. For the mammary fat pad implantation, 1 × 10^5^ 4T1 cells in a volume of 10ul were implanted into the mammary fat pad of 6–8-week-old female Balb/c mice following an established protocol [25]. Primary tumors were measured with calipers, and tumor volumes were calculated by the formula (*S* × *S* × *L*) × 0.52, where S and L are the short and long dimensions, respectively [30]. Tumors and blood samples were collected at indicated time point. Tumors were surgically removed and either fixed for immunohistochemistry staining analysis or dissociated with type I collagenase followed by DNase I treatment. Red blood cells were removed from the dissociated tumors followed by flow cytometry analysis. Blood was collected from heart ventricle followed by removing red blood cells with ACK lysis buffer. Cells were pelleted and suspended in FACS buffer followed by flow cytometry analysis. Mice were sacrificed and the lungs were surgically removed followed by staining with the Bouin's solution (Sigma). The lung metastasis was determined by counting the cancer nodules on the lungs. Alternatively, total RNA was extracted from the lungs and qRT-PCR was performed to quantify the labelled cancer cells with reference genes.

For tail vein injection, 1 × 10^6^ of MDA-MB-231 cells, 5 × 10^4^ of 4T1 cells, or 1 × 10^5^ of 4TO7 cells were administrated through the tail vein of female nu/nu mice for MDA-MB-231 cells and female Balb/c mice for 4T1 and 4TO7 cells, respectively. Lung colonization was quantified as described above.

For tumor formation of MDA-MB-231 cells, 1 × 10^6^ cells were injected subcutaneously with matrigel to female nu/nu mice. Tumor progression was determined as described above.

### Database analysis

Three databases of breast cancer patients were used in this study: TCGA, the NCBI GEO database (GSE22220), and Metabric (EGAS00000000122) [58]. Kaplan-Meier survival curve analyses were carried out using log-rank tests in GraphPad Prism (GraphPad Software). Patients were divided into two groups based on median miRNA expressin of all patients, and subsequently different subtypes of breast cancer were further analyzed.

## SUPPLEMENTARY MATERIALS FIGURES AND TABLES


