# Supplementary material for: MiR-148a functions to suppress metastasis and serves as a prognostic indicator in triple-negative breast cancer
Source: Oncotarget. 2016 Mar 7;7(15):20381–94. doi: 10.18632/oncotarget.7953 (PMC4991462; doi:10.18632/oncotarget.7953)
Supplement: Supplementary file 1 [file oncotarget-07-20381-s001.pdf]

MiR-148a functions to suppress metastasis and serves as a prognostic indicator in triple-negative breast cancer

Supplementary Materials

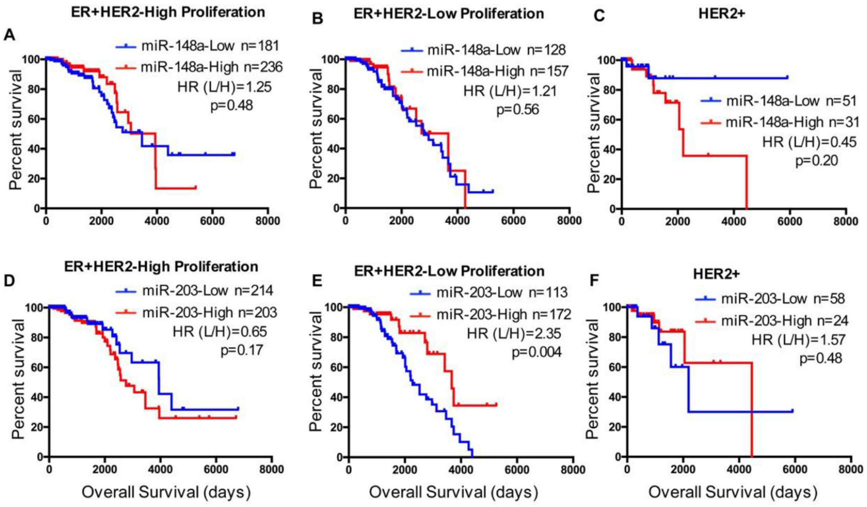

Supplementary Figure S1: Correlation analysis of patient overall survival to the expression of miR-148a. Kaplan-Meier curves for overall survival for high and low expression of miRNAs, miR-148a (C) and miR-203 (D), in three subtypes of breast cancer, ER+HER2- High proliferation (**A** and **D**), ER+HER2- Low proliferation (**B** and **E**), and HER2+ (**C** and **F**). Data was extracted from ***TCGA***. *P* values were calculated with Log-rank (Mantel-Cox) test. Hazard ratios were calculated using the method of Mantel-Haenszel.

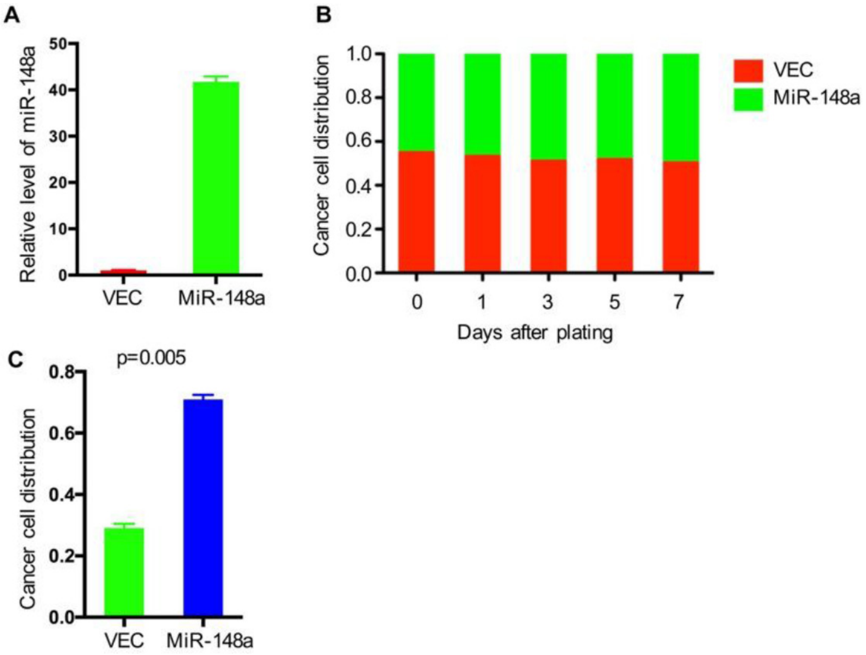

Supplementary Figure S2: Characterization of 4T1 cells with expression of fluorescent proteins and miR-148a. (**A**) The expression of miR-148a was confirmed in 4T1 cells with iRFP (VEC) or GFP-miR-148a (miR-148a) and normalized to VEC cells. (**B**) competitive growth of VEC (iRFP) and miR-148a (GFP) cells was evaluated using *in vitro* co-culture followed by FACS. (**C**) Distribution of cancer cells (VEC-GFP vs miR-148a-BFP) in primary tumors at day 7. *P* value was calculated with two-tailed unpaired *t* test ($n = 3$). Error bar (SE).

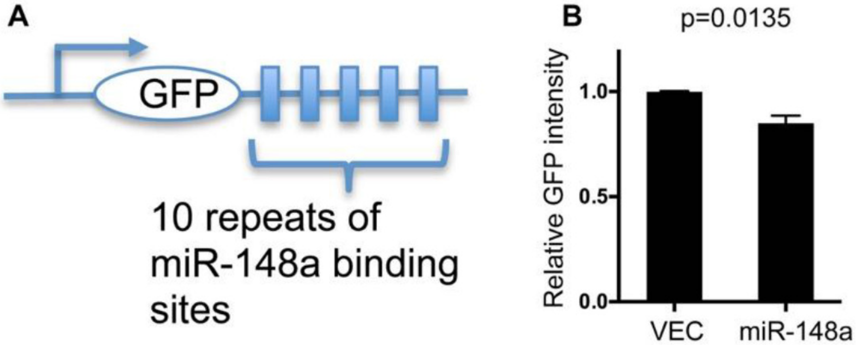

Supplementary Figure S3: Validation of the effects of the miR-148a sponge. (**A**) an illustration of miR-148a sponge design, reporter gene GFP followed by 10 repeats of DNA fragments which are designed to bind miR-148a. (**B**) The miR-148a sponge was co-transfected with control or miR-148a into 293T cells. iRFP was used as reference for normalization. GFP and iRFP intensity was determined by flow cytometry. The reduction of normalized GFP intensity indicates the effectiveness of the miR-148a sponge.

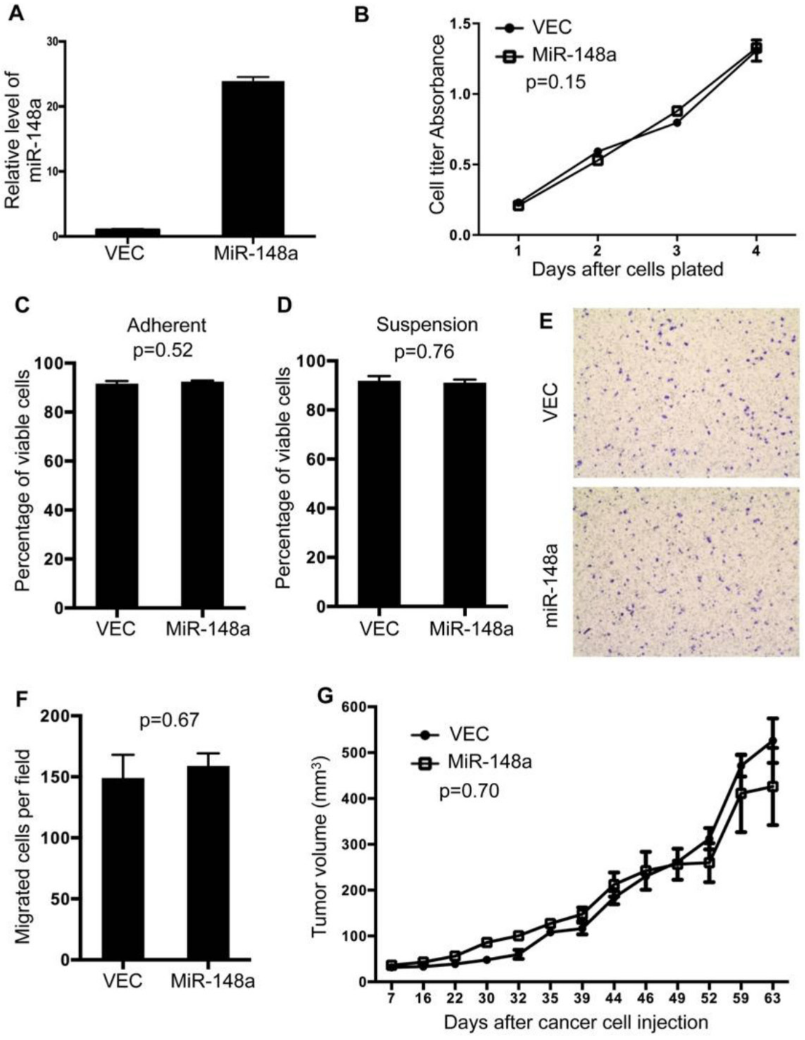

Supplementary Figure S4: Characterization of MDA-MB-231 cells with overexpression of miR-148a. (**A**) The expression of miR-148a was determined in MDA-MB-231 cells with overexpression of miR-148a (miR-148a) and normalized to control cells (VEC). (**B–F**) MDA-MB-231 cells with over expression of miR-148a (miR-148a) and control cells (VEC) were examined for growth (B), viability (C), anoikis resistance (D), and migration (E and F). Error Bars indicate Standard Errors (n = 3). P value was calculated with two-way ANOVA (B) or unpaired, two-tailed t test (C, D, and F). (E) Representative pictures of migrated cells are shown. (F) (**G**) Tumor progression was monitored by measuring tumor size at indicated time points. P values were calculated with two-way ANOVA. Error Bars indicate Standard Errors (n = 5).

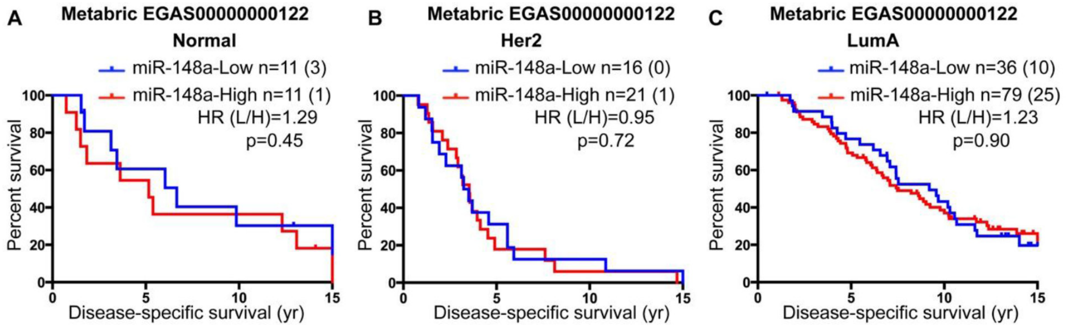

Supplementary Figure S5: Correlation analysis of patient survival to the expression of miR-148a. Normal subtype (**A**) Her2 subtype (**B**) and LumA subtype of patients with metastasis were analyzed with Kaplan-Meier curves for high and low expression of miR-148a. Data was extracted from ***METABRIC data*** [55]. Survival time was cropped at 15 years. *P* values were calculated with Log-rank (Mantel-Cox) test. Hazard ratios were calculated with the method of Mantel-Haenszel). Numbers of patients censored are indicated in (**C**).

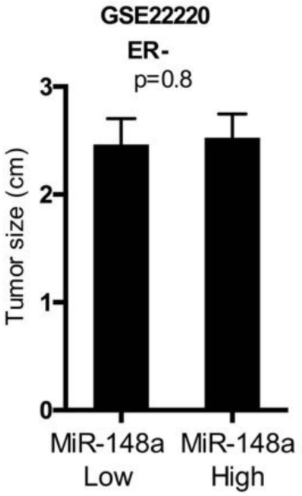

Supplementary Figure S6: Correlation analysis of patient tumor size to the expression of miR-148a. Data was extracted from the database **GSE22220.** Patients were categorized as high or low expression of miR-148a (n = 40) and patient tumor sizes were plotted. *P* value was calculated with two-tailed unpaired *t* test. Error bars (SE).

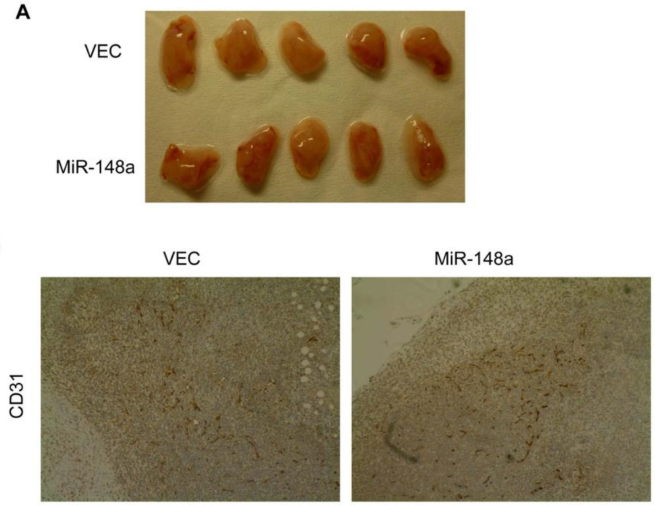

Supplementary Figure S7: Angiogenesis in primary tumor. Primary tumors arisen from 4T1 cells with control vector or miR-148a overexpression were collected at 1 week (**A**) and two weeks (**B**) after cancer cell implantation. (A) Angiogenesis was visualized by the redness of the tumor surface. (B) Tumor samples were processed for IHC staining with anti-CD31 to examine angiogenesis.

Supplementary Table S1: Summary of candidate target genes of miR-148a

Gene symbol	ΔΔCt gene array	Miranda prediction	Targetscan prediction	Correlation with miR-148a	Subcellular localization
INHBB	−3.0	yes	yes	yes	Secreted
SNAP91-1	−2.2	yes	no	no	Cytoplasma
SNAP91-2	−1.8	yes	no	no	Cytoplasma
TXNIP	−1.3	yes	yes	yes	Nucleus
RPXS6KA2	−1.2	yes	no	yes	Nucleus
WNT1	−1.2	yes	yes	yes	Secreted
PIK3C2B	−1.1	yes	no	yes	Nucleus, Cytoplasma, Cell membrane
PTGS1	−1.1	yes	no	yes	Nucleus and cytoplasma
OXTR	−1.0	yes	no	yes	Nucleus and cytoplasma
BTBD3	−0.9	yes	yes	no	Nucleus and cytoplasma
MITF	−0.8	yes	yes	yes	Nucleus
EPHA4	−0.8	yes	no	no	Cell membrane
NRP1	−0.7	yes	yes	yes	Cell membrane
FLOT2	−0.7	yes	no	no	Cell membrane
CD247	−0.6	yes	no	no	Cell membrane

A list of genes validated with down regulation by enhanced overexpression of miR-148a. Only genes with ΔΔCt less than −0.5 are listed.

Supplementary Table S2: Percentage of breast cancer patients with different subtypes analyzed in groups separated by tumor grade and expression of miR-148a

Diagnosis	Subtypes	miR-148a Low	miR-148a High
Grade 1	Basal	0	1
	LumA	22	60
	LumB	1	7
	Her2	0	1
	Normal	8	4
	Total	31	73
Grade 2	Basal	14 (6.4%)	5 (1.8%)
	LumA	110 (50.5%)	197 (70.9%)
	LumB	72 (33%)	53 (19.1%)
	Her2	8 (7.3%)	11 (4%)
	Normal	14 (6.4%)	12 (4.2%)
	Total	218 (100%)	278 (100%)
Grade 3	Basal	103 (28.3%)	50 (19.3%)
	LumA	57 (15.7%)	85 (32.8%)
	LumB	148 (40.7%)	60 (23.2%)
	Her2	33 (9.1%)	50 (19.3%)
	Normal	23 (6.2%)	14 (5.4%)
	Total	364 (100%)	259 (100%)

Patients were categorized with expression levels of miR-148a as low or high and then stratified by diagnosed tumor grade. Patient numbers were counted in each subtype group and percentages were calculated in particular grade with miR-148a low or high. Data was extracted from ***METABRIC data*** [55].

Supplementary Table S3: Analysis of the expression of miR-148a in primary tumors in breast cancer patients with or without metastasis

Database	Met	Non-met	*p* value
Metabric EGAS00000000122	1928 ± 77.20 (*n* = 353)	2214 ± 54.73 (*n* = 933)	0.0046
GSE22220	28536 ± 416.5 (*n* = 79)	30014 ± 220.2 (*n* = 131)	0.0007
TCGA	35827 ± 5874 (*n* = 13)	52635 ± 2302 (*n* = 412)	0.197

Data was extracted from three databases (**METABRIC data, GSE22220, and TCGA**) to analyze the expression of miR-148a (Mean ± SEM) in patients with or without metastasis. *P* values were calculated with unpaired, two-tailed *t* test**.**
